# Thiourea-Capped Nanoapatites Amplify Osmotic Stress Tolerance in *Zea mays* L. by Conserving Photosynthetic Pigments, Osmolytes Biosynthesis and Antioxidant Biosystems

**DOI:** 10.3390/molecules27185744

**Published:** 2022-09-06

**Authors:** Sana Faryal, Rehman Ullah, Muhammad Nauman Khan, Baber Ali, Aqsa Hafeez, Mariusz Jaremko, Kamal Ahmad Qureshi

**Affiliations:** 1 Department of Chemical and Life Sciences, Qurtuba University of Science and Information Technology, Peshawar 25124, Pakistan; 2 Department of Botany, University of Peshawar, Peshawar 25120, Pakistan; 3 Department of Botany, Islamia College Peshawar, Peshawar 25120, Pakistan; 4 Department of Plant Sciences, Quaid-i-Azam University, Islamabad 45320, Pakistan; 5 Smart-Health Initiative (SHI) and Red Sea Research Center (RSRC), Division of Biological and Environmental Sciences and Engineering (BESE), King Abdullah University of Science and Technology (KAUST), Thuwal 23955, Saudi Arabia; 6 Department of Pharmaceutics, Unaizah College of Pharmacy, Qassim University, Unaizah 51911, Saudi Arabia

**Keywords:** thiourea, nanoapatite, characterization, APX, SOD, POD, osmolytes

## Abstract

Salinity is one of the most prevalent abiotic stresses which not only limits plant growth and yield, but also limits the quality of food products. This study was conducted on the surface functionalization of phosphorus-rich mineral apatite nanoparticles (ANPs), with thiourea as a source of nitrogen (TU–ANPs) and through a co-precipitation technique for inducing osmotic stress tolerance in *Zea mays*. The resulting thiourea-capped apatite nanostructure (TU–ANP) was characterized using complementary analytical techniques, such as EDX, SEM, XRD and IR spectroscopy. The pre-sowing of soaked seeds of *Zea mays* in 1.00 µg/mL, 5.00 µg/mL and 10 µg/mL of TU–ANPs yielded growth under 0 mM, 60 mM and 100 mM osmotic stress of NaCl. The results show that Ca and P salt acted as precursors for the synthesis of ANPs at an alkaline pH of 10–11. Thiourea as a source of nitrogen stabilized the ANPs’ suspension medium, leading to the synthesis of TU–ANPs. XRD diffraction analysis validated the crystalline nature of TU–ANPs with lattice dimensions of 29 nm, calculated from FWHM using the Sherrer equation. SEM revealed spherical morphology with polydispersion in size distribution. EDS confirmed the presence of Ca and P at a characteristic KeV, whereas IR spectroscopy showed certain stretches of binding functional groups associated with TU–ANPs. Seed priming with TU–ANPs standardized germination indices (*T*50, *MGT*, *GI* and *GP*) which were significantly declined by NaCl-based osmotic stress. Maximum values for biochemical parameters, such as sugar (39.8 mg/g at 10 µg/mL), protein (139.8 mg/g at 10 µg/mL) and proline (74.1 mg/g at 10 µg/mL) were recorded at different applied doses of TU–ANP. Antioxidant biosystems in the form of EC 1.11.1.6 catalase (11.34 IU/g FW at 10 µg/mL), EC 1.11.1.11 APX (0.95 IU/G FW at 10 µg/mL), EC 1.15.1.1 SOD (1.42 IU/g FW at 5 µg/mL), EC 1.11.1.7 POD (0.43 IU/g FW at 5 µg/mL) were significantly restored under osmotic stress. Moreover, photosynthetic pigments, such as chlorophyll A (2.33 mg/g at 5 µg/mL), chlorophyll B (1.99 mg/g at 5 µg/mL) and carotenoids (2.52 mg/g at 10 µg/mL), were significantly amplified under osmotic stress via the application of TU–ANPs. Hence, the application of TU–ANPs restores the growth performance of plants subjected to induced osmotic stress.

## 1. Introduction

Maize is grown worldwide and is the third major crop after rice and wheat, named as “king of grain crops” [1]. The USA, China, Brazil and Argentina share two-thirds of the global maize production alone [2]. In Pakistan, *Zea mays* is cultivated on an area of about 896 thousand hectares with a yearly mass production of 4.9 million tons of grains [3]. It is widely cultivated in KP and Punjab, contributing about 98% of the total production of Pakistan [4]. Salinity affects the morphology, physical appearance and biochemical composition of maize, including germination and photosynthesis, and thus affects the development of the plant by declining the leaf area, photosynthetic pigments and stomatal function [5]. In Pakistan, about 6.62 million hectors, accounting for eight percent of the total cultivable land, is adversely affected by salinization [6]. Thus, Pakistan stands at eighth place in the world for high saline land, due to which PKR 15 to 55 billion (about 0.6% of the total GDP) annual economic loss is estimated as a result of poor production of crops and crop products [7].

Recent trends in agriculture have validated the use of plant growth regulators, including thiourea, to surge the growth and yield of crop plants. Thiourea (TU) not only provides a nitrogen source, but also augments tolerance against various abiotic stresses due to its high degree of solubility and rapid absorption into biosystems [8]. Earlier studies have shown that thiourea application significantly improved the photosynthetic efficiency and growth of wheat crop [9,10]. TU has been recognized as an effective osmoprotectant to safeguard the crops from the detrimental effects of various abiotic stresses, including osmotic stress and thermal stress. TU-based tolerance is credited with the activation of biosystems for an enhanced uptake of nutrients, synthesis of osmolytes and scavenging ROS, as well as triggering antioxidation biosystems [11,12]. However, it is estimated that 75% of the nitrogen source, when directly applied to soil, is lost due to many reasons, including nitrate leaching and soil erosion [13]. Thus, farmers are compelled to use more fertilizer for better plant growth, which is not only costly, but also causes negative impacts on the environment [14,15]. In order to cope with this problem, scientists worldwide use nanotechnology, which is a vital process for forming novel systems at nanoscale, possessing some novel properties that promote plant productivity and reduce environmental pollution, which are released in soil in a controlled manner [16].

Nanoparticles have unique physicochemical properties, which are used for different purposes in life sciences, chemical engineering, medicines and agriculture [17]. Hence, the present study was designed with the aim of synthesizing thiourea-based apatite nanoparticles (TU–ANPs), using urea both as a source of nitrogen and to cope with NaCl-based osmotic stress in *Zea mays* via germination and seedling foliar application.

## 2. Results

### 2.1. Characterization of TU–ANPs

**Figure 1:**
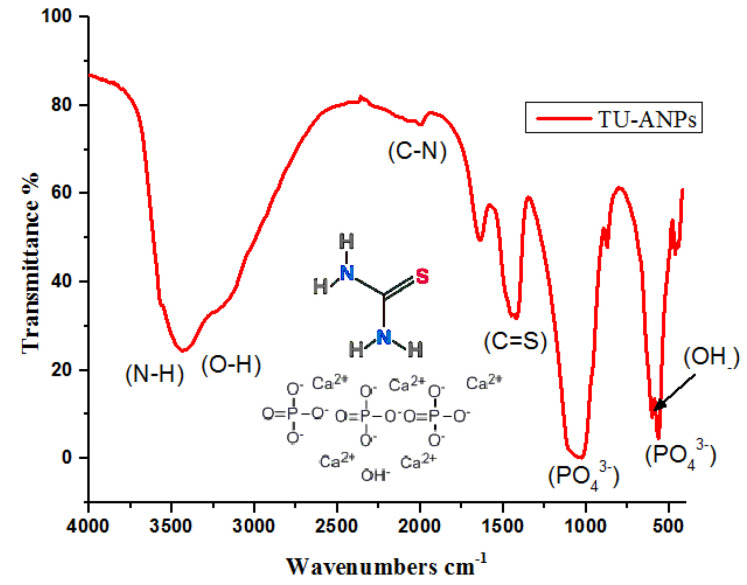
Infrared spectroscopy of TU-ANPs representing various vibrational stretches and bending of functional groups associated with thiourea and apatite.

**Figure 2:**
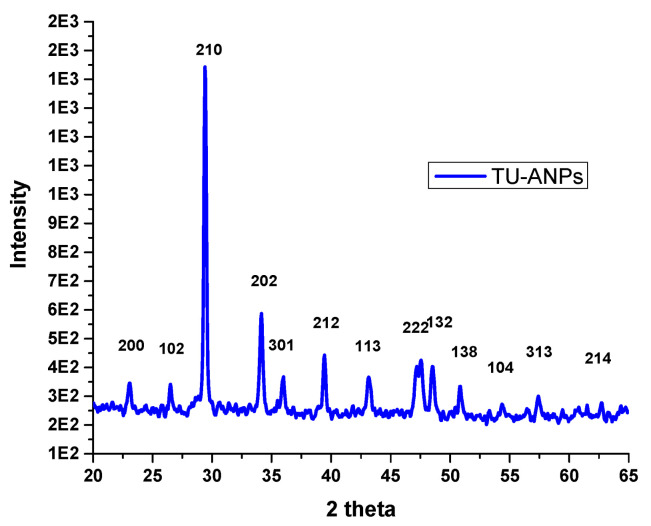
X-ray diffraction analysis of TU–ANPs showing intensities at different two theta levels matching a standard spectra of apatite with JCPDS: 00-009-0432.

**Figure 3:**
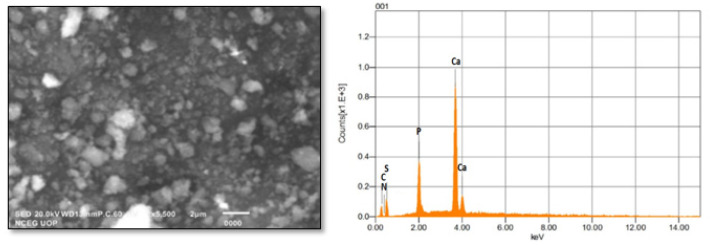
SEM–EDX analysis of TU–ANPs showing intense agglomeration due to Ostwald ripening where the size ranges from 40 nm to 220 nm, and characteristic signals of P and Ca of apatite at given KeV.

FTIR spectral analysis (Figure 1) revealed that: IR absorption bands located at 3428, 3240, 1999, 1427 wavenumbers cm^−1^ correspond to N-H stretching; and O-H, C-N and C=S are associated with thiourea and vibrational bending at 1032 and 520 wavenumbers cm^−1^, representing PO_4_^3−^ characteristics in the apatite-validating capping of thiourea on the surface of ANPs (Figure 1). The FTIR study validates the fact that displacement of the C=S stretch is indicative of the binding of thiourea via this group to the core ANPs, hence functionalizing the surface of ANPs and preventing an agglomeration of particles. Consequently, the medium is stabilized. The XRD pattern showed intense diffraction peaks at 2θ; 31.77, 32.90, 35.95, 45.47, 49.23, 57.80, 62.85, 68.49 and 25.96 and matched with JCPDS: 00-009-0432 as the standard diffraction spectra of apatite (Figure 2). SEM images revealed the spherical-to-oval morphology of TU–ANPs with an intense agglomerated Ostwald ripening, where the size ranged between 40 nm to 220 nm in diameter (Figure 3). EDX spectra showed the presence of calcium (Ca) at 3.9 and 4.0 KeV, while phosphorus intensity was observed around 2.0 KeV, which is characteristic of ANPs. The presence of nitrogen, carbon and sulfur in traces are due to the capping of thiourea on the surface of ANPs (Figure 3).

### 2.2. Effect of TU–ANPs on Germination Indices and Vegetative Performance

**Figure 4:**
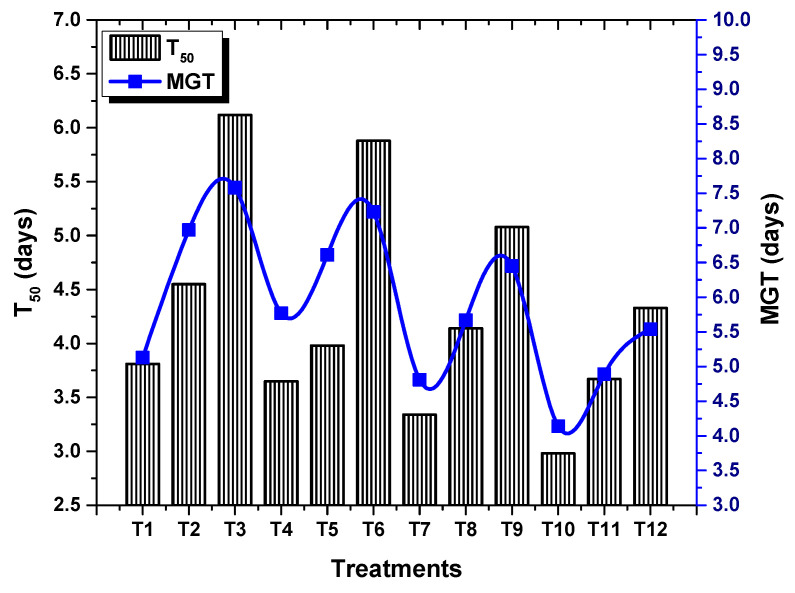
Effect of TU–ANPs on mean germination time (*MGT*) and time to 50% germination (*T*50) of *Zea mays* under various levels of NaCl-based osmotic stress.

**Figure 5:**
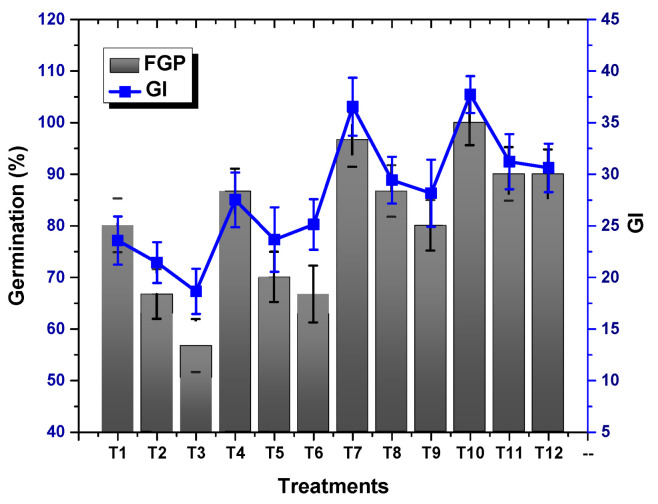
Effect of TU–ANPs on germination index (*GI*) and germination percentage (*GP*) of *Zea mays* under various levels of NaCl-based osmotic stress.

**Figure 6:**
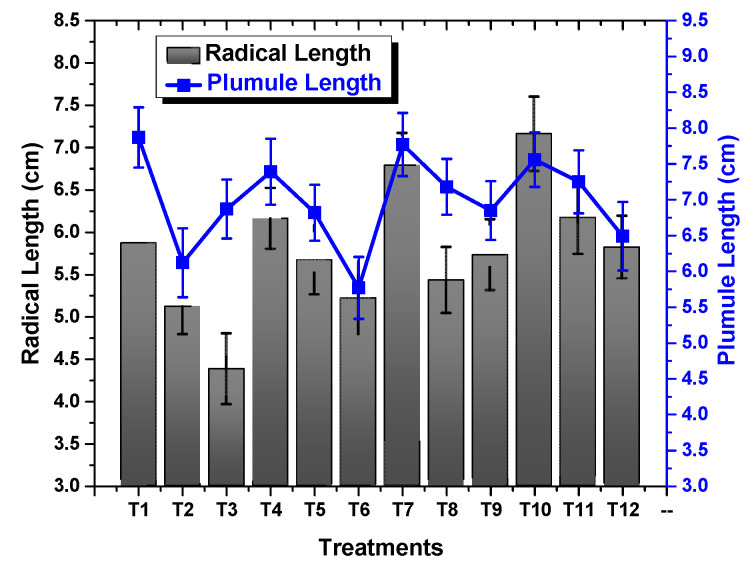
Effect of TU–ANPs on seedling growth (plumule and radical) of *Zea mays* under various levels of NaCl-based osmotic stress.

ANOVA results revealed a significant (*p* < 0.05) effect of TU–ANPs on *GP*, *MGT*, *GI* and *T*50. Seed grown under osmotic stress of 100 mM NaCl delayed the onset of germination to 6.12 days, compared to the non-saline condition (3.18). Seeds primed with 10 µg/mL of TU–ANPs induced early germination with a *T*50 value of 2.98 days under the non-saline condition whereby, under 100 mM salt stress, 3.67 days were taken to reach 50% germination (Figure 4). Similarly, *MGT* and *GI* values were also significantly stabilized by TU–ANPs under NaCl-induced osmotic stress. A maximum *GP* value of 100% was exhibited by seeds primed at 10 µg/mL, followed by a 96.67% *GP* value at 5 µg/mL, where a minimal *GP* of 66.67% was exhibited by hydro-primed seeds grown under 100 mM NaCl-based osmotic stress (Figure 5). Moreover, a significant decline in seedling growth under osmotic stress was reinstated via the application of TU–ANPs (Figure 6). The mean maximum root length (7.1 cm) was recorded for plants treated with 10 µg/mL of TU–ANPs under the non-saline condition, followed by plants (7.0 cm) treated with 5 µg/mL of TU–ANPs as shown in Table 1. The minimal root length (5.5 cm) under osmotic stress of 100 mM NaCl was significantly elevated to 6.1 cm when primed with 5 µg/mL of TU–ANPs (Table 1). Similarly, data summarized in Table 1 reveals a highly significant (*p* < 0.05) dose-dependent decline in leaf area, leaf fresh and dry biomass, due to NaCl-induced osmotic stress. Foliar application of TU–ANPs at a dose of 5.0 µg/mL exhibited 1.5 g/leaf FW, 0.22 g/leaf DW and 40.87 cm^2^ leaf area in plants grown under the highest experimental dose of osmotic stress. Moreover, maximum shoot length (8.7 cm) was also recorded in plants with a foliar application of 5 µg/mL under 60 mM of salinity, followed by hydro-primed plants (8.5 cm) of the non-saline condition (Table 1).

### 2.3. Effect of TU–ANPs on Photosynthetic Pigments and Osmolytes

**Figure 7:**
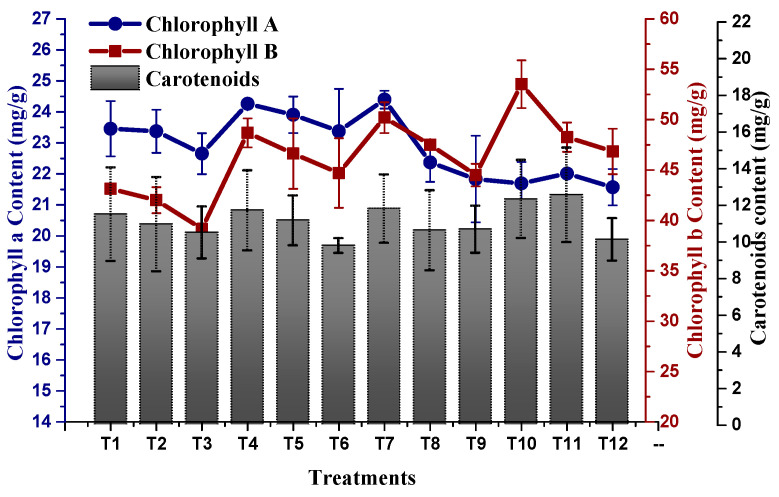
Effect of TU–ANPs on photosynthetic contents (chlorophyll a, b and carotenoids) of *Zea mays* under various levels of NaCl-based osmotic stress.

**Figure 8:**
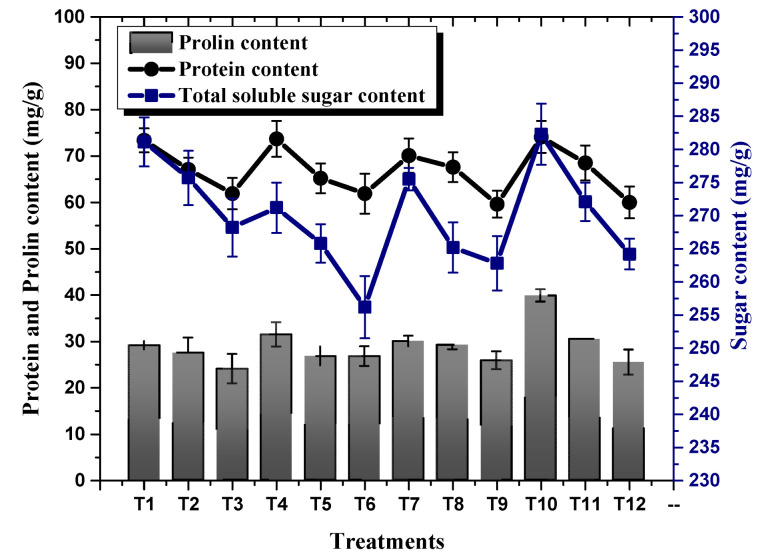
Effect of TU–ANPs on osmolytes content (protein, proline and soluble sugar) of *Zea mays* under various levels of NaCl-based osmotic stress.

Plants exposed to NaCl-based osmotic stress significantly declined their photosynthetic pigments (Chl a, Chl b and carotenoids). A minimal chlorophyll b content of 39.15 mg/g was recorded in plants subjected to 100 mM NaCl-based osmotic stress, whereby a maximum chlorophyll b content of 53.51 mg/g was recorded in plants treated with 10 µg/mL of TU–ANPs via the foliar route grown under the non-saline condition. This was followed by 50.20 mg/g in plants treated with 5 µg/mL of TU–ANPs. Similarly, chlorophyll a content in plants was also subjugated to decline due to osmotic stress in a dose-dependent manner (Figure 7). An almost similar pattern of values was also reported for carotenoid levels, where its highest value of 12.54 mg/g was recorded in plants with under 100 mM of NaCl-based salinity and which were treated with 10 µg/mL of TU–ANPs. Plants with under 100 mM NaCl-based osmotic stress were observed with a mean chlorophyll a level of 22.65 mg/g, whereby in non-saline plants, the value 23.45 mg/g was recorded for chlorophyll a content. Application of TU–ANPs at an optimal experimental dose (5 µg/mL) elevated the chlorophyll a level to 24.39 mg/g; meanwhile, at a higher dose of TU–ANPs, a decline (21.83 mg/g) in chlorophyll a levels was reported (Figure 7). Likewise, foliar application of TU–ANPs in maize plant under osmotic stress significantly affected levels of osmolytes (protein, sugar and proline). A total of 39.8 mg/g of protein was recorded in plants treated with 10 µg/mL of TU–ANPs under a non-saline condition, whereas 24.0 mg/g of leaf protein content was recorded in untreated plants (hydro-applied) under 100 mM of NaCl-based osmotic stress. The results obtained clearly indicate that the application of TU–ANPs optimized the level of protein, hence amplifying the growth performance of plants under osmotic stress (Figure 8). Likewise, proline content (74.1 mg/g) was also ameliorated via the application of 10 µg/mL of nanoparticles, compared to (60.0 mg/g) plants subjected to hydro-application and 100 mM NaCl-based osmotic stress (Figure 8). However, total soluble sugar was non-significantly affected by osmotic stress, whereby its highest value was obtained at T10 (10 µg/mL NPs), followed by T1 (hydro-applied) and T7 (5 µg/mL NPs). Meanwhile, the lowest level (256.2 mg/g) was reported at T6 (1 µg/mL NPs + 100 mM NaCl).

### 2.4. Effect of TU–ANPs on Antioxidation Biosystem

**Figure 9:**
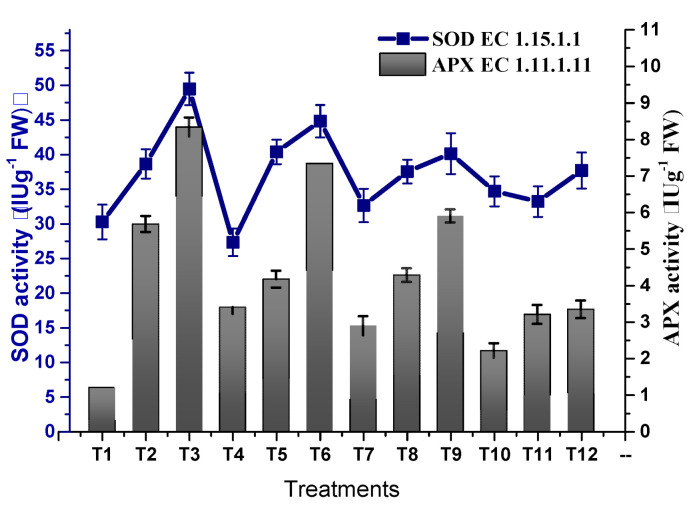
Effect of TU–ANPs on activities of superoxide dismutase (SOD) and ascorbate peroxidase (APX) of *Zea mays* under various levels of NaCl-based osmotic stress.

**Figure 10:**
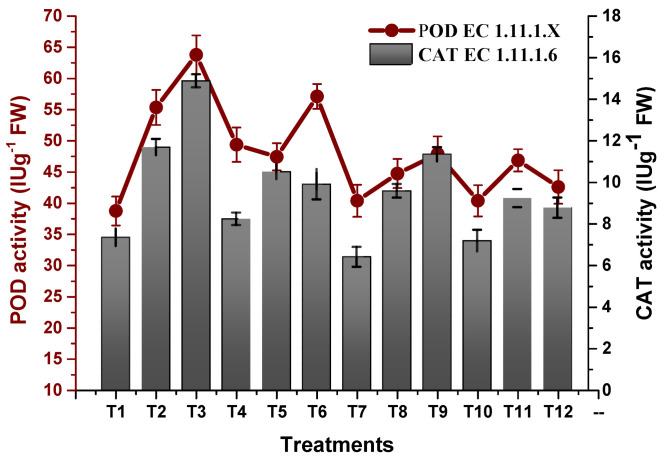
Effect of TU–ANPs on activities of peroxidase (POD) and catalase (CAT) of *Zea mays* under various levels of NaCl-based osmotic stress.

Osmotic stress inducing oxidative stress with the production of oxidative free radicals led to a decline in plant growth. TU–ANPs significantly coped with free radicals by triggering the antioxidative biosystems of plants, hence optimizing the activity of antioxidant enzymes. The activity of SOD was significantly enhanced from 30.28 IU/G FW in non-saline plants to 49.48 IU/g FW in plants subjected to 100 mM NaCl-induced osmotic stress. Foliar application of TU–ANPs at 10 µg/mL significantly reinstated the activity of SOD to 37.7 IU/g FW in plants under the highest experimental osmotic stress (Figure 9). A similar trend in the incline of POD activity (63.79 IU/g FW) was recorded at T3 (100 mM NaCl) against 38.76 IU/g FW at T1 (non-saline). The application of TU–ANPs at T7 and T10 significantly stabilized POD activity to 47.90 and 42.61 IU/g FW against the 63.79 IU/g FW value at T1 in plants under elevated osmotic stress. A similar trend in the elevation of APX and CAT activities was reported under osmotic stress, whereby TU–ANPs standardized their respective activities to 3.33 and 8.76 IU/g FW (T12) against 5.67 and 11.68 IU/g FW in hydro-applied plants (T3) under elevated (100 mM NaCl) osmotic stress (Figure 10).

## 3. Discussion

NaCl-based osmotic stress not only causes a reduction in *GP* but also delays the time to germination (*MGT* and *T*50). This is due to its negative effect on mitotic cell division and cell elongation, altering enzyme activity particularly associated with protein synthesis, as well as growth hormones [18]. Figure 4, Figure 5 and Figure 6 show that the application of TU–ANPs significantly stabilize germination indices by not only providing minerals source of calcium, phosphorus and nitrogen, but also by triggering biosynthetic pathways associated with sugar phosphates, nucleotides, NADH, amino acids and growth hormones, such as IAA. Meanwhile, thiourea is also known for breaking environmentally imposed seeds, as well as innate seeds and innate bud dormancy, hence resulting in the early onset of seed germination and the establishment of seedling growth [19,20].

Plant agronomic traits were significantly declined by soil salinity due to nutritional imbalances in plants caused by salt ions through competitive absorption distribution and the transport of micro- and macro-minerals [21]. Salinity primarily affects the bioavailability of nitrogen by inhibiting root nodulation, phosphorus by inhibiting its uptake, and potassium by altering its uptake and transportation. Besides the nutrient availability, salt also induces oxidative stresses through the production of peroxides (H_2_O_2_), nascent oxygen, hydroxyl free radicals (HO*) and superoxide anion (O^2−^) which are not only cytotoxic, but also cause damages to vital biomolecules (lipids, protein, DNA and carbohydrates) through oxidation, hence effecting the overall growth performance of plants [22].

Similarly, high soil–salt content inhibits the activity of RUBisCO and ATPase, causing the reduction in CO_2_ assimilation, and consequently reducing the efficiency of thylakoids and mitochondrial electron transport [23]. Plant agronomic parameters were significantly influenced by the application of TU–ANPs, salt stress and their interactions. The application of TU–ANPs (1 µg/mL, 5 µg/mL and 10 µg/mL), particularly in non-saline conditions, caused a significant increase in plant height, dry and fresh weight, shoot length, shoot fresh and dry weight, root length and root fresh and dry weight, hence the use of nanoparticles enhanced the growth of the maize plant by limiting the negative effects of salt stress. Nano-fertilizers extend the duration of nutrient release to the plant, enhance the absorption of nutrients, increase the accumulation of nitrogen in the leaf to support leaf development and consequently balances the nutrient loss due to excess salts [24]. Mitigating crop allometric traits, such as root and shoot length, leaf area, and fresh and dry biomass by thiourea, might be due to the improved translocation of photosynthates. It was reported previously that foliar application of thiourea significantly augmented the growth-related parameters in *Vicia faba* [25]. Moreover, nano-thiourea (TU–ANPs) modulated plants’ vegetative growth under salinity via the up-regulation of phytohormones synthesis, particularly auxins, regulating gene expression at the post-transcriptional stage and co-coordinating the regulation of microRNA expression [26].

Additionally, nano-fertilizer encouraged enzyme activities in plants; as a result, plant cells preserved from injury of reactive oxygen species (ROS) [27]. In addition, the application of TU–ANPs provides calcium- and phosphorus-enriched supplements, which reduces the efflux of Kþ and influx of Naþ by stalling nonselective Na^+^/K^+^ channels, thus coping with the detrimental effects of salinity [28]. Ca is also an obvious component of multiple plant signaling pathways, remarkably including the SOS-regulatory pathway, which manages the sequestration of Naþ from the cytosol into the vacuole [29]. Moreover, reduction in photosynthetic pigments in plants under salt stress is of common occurrence that has been reported in a wide variety of plants [30]. Salinity tends to inhibit the production of chlorophyll through the activation of the chlorophylytic enzyme (chlorophyllase) and chloroplast membrane degradation, the production of ROS and the inactivation of the Rubisco enzyme [31,32]; thus, a decline in the pigment of leaves damages the structure of the leaf by minimizing the chloroplast [33,34]. Application of thiourea-based ANPs improves the efficiency of photosystem I and II and boosts the plants antioxidant capabilities [35]. A number of soluble sugars are also disturbed by applying salinity, but the application of nanoparticles of silver improves the sugar content.

Similarly, proline content in plant tissues is both a reflection and a measure of stress-induced damage at the cellular level. Accumulation of proline under stress protects the cell by balancing the osmotic strength of cytosol with that of the vacuole and external environment [36]. Our results reveal that exogenous application of TU–ANPs elevated proline levels by providing a nitrogen source for the biosynthesis of amino acids, including proline, which not only acts as osmolyte, but also interacts with other cellular macro-molecules while stabilizing their structure and function [37,38].

The present study imitated an increase in activities of antioxidant enzymes (POD, APX and CAT, etc.) in response to NaCl-based osmotic stress due to the production of ROS (H_2_O_2_, HO* and O^2−^) [39]. Our results are in agreement with the findings of Zainab et al. [36], who also reported an elevation in the activity of antioxidant enzymes in pearl millet under 120 and 150 mM applied NaCl stress. Results summarized in Figure 9 and Figure 10 show a significant stabilization of the activity of antioxidant enzymes in plants under stress by applying TU–ANPs, which may be attributed to the eliciting effect of thiourea by providing the amino source (nitrogen) to synthesize different antioxidants, including amino acids, glutathione, etc., as well as scavenging ROS through its thiol group.

## 4. Material and Methods

### 4.1. Synthesis and Characterization of TU–ANPs

While following the biomimetic precipitation procedure of Paz et al. [40] with slight modifications, TU–ANPs were synthesized. Initially, 2 mM aqueous Ca^+2^ (calcium salt) and aqueous Na_2_HPO_4_ (2 mM) in NaHCO_3_ (2 mM) were mixed drop-wise with a continuous stirrer at 37 °C and pH 7.4, until the appearance of suspended nanoparticles. The resulting ANPs were centrifuged, washed and resuspended in the nitrogen source (thiourea) for surface functionalization for 72 h under vigorous stirring. The resulting TU–ANPs were centrifuged at 8000 rpm, washed, freeze-dried and subjected to contemporary characterization, such as IR spectroscopy, SEM, EDX and XRD analysis using Oxford Inca 200 SEM instrument equipped with a Thermo EDX attachment and JEOL JDX 3532 X-ray diffractometer at the University of Peshawar.

### 4.2. Plant Material and Growth Conditions

An experiment under laboratory conditions was performed using a randomized complete block design at the Department of Botany, University of Peshawar, Pakistan, to determine various germination indices. Primed seeds of *Zea mays* (SB-989 variety) in dH_2_O, 1 µg/mL, 5 µg/mL and 10 µg/mL of TU–ANPs were sown in Petri plates with double folds of Whatman filter paper under 0 mM, 60 mM and 100 mM of NaCl-induced osmotic stress with three replicates (30 seeds) in each treatment condition at 25 °C. The time to 50% germination (*T*50), mean germination time (*MGT*), percent germination (PG) and germination index were calculated using the Equations as follows:

(1) $$MGT=\frac{\sum \left(Ni\times Ti\right)}{N}$$

(2) $$GI=\frac{G1}{D1}+\ldots +\frac{Gf}{Df}$$

(3) $$T50=Ta+\frac{\left(\frac{N}{2}-Na\right)\left(Tb-Ta\right)}{\left(Nb-Na\right)}\;$$

(4) $$GP=\frac{Gt}{Nt}$$

where *Ni* is the number of seeds germinated on the day; (*Ti*) is the time (days); *G*1 is the germinated seeds on the first day (*D*1); *Gf* is the final germinated count on the final day (*Df*); *N* is the number of seeds germinated on the final day; *Na* and *Nb* are the respective cumulative germination at *Ta* and *Tb* (times in days); *Gt* is the total number of germinated seeds; and *Nt* is the total number of seeds.

Similarly, the field experiment was performed using a randomized complete block design. Seeds of *Zea mays* in primed in dH_2_O, 1 µg/mL, 5 µg/mL, and 10 µg/mL of TU–ANPs were sown in pots and filled with loam and ten seeds each at the Department of Botany, University of Peshawar, Pakistan (34.0086° N, 71.4878° E). After completion of the seed germination (7 days), pots were placed under osmotic stress using 0 mM, 60 mM and 100 mM of NaCl solution. The stress was maintained with a weekly application of the mentioned doses of NaCl. Plants were, respectively, treated with dH_2_O, 1 µg/mL, 5 µg/mL and 10 µg/mL of TU–ANPs via the foliar route with an interval of 7 days for 6 weeks. Upon the completion of the seventh week, plants were harvested and initially subjected to data collection for vegetative growth parameters (shoot length, root length, number of leaves, etc.). Fresh plant specimens were also subjected to data collection in order to quantify photosynthetic pigments, osmolytes and antioxidant enzymes.

### 4.3. Physiological and Biochemical Analysis

Photosynthetic pigments were quantified through a UV–Vis spectrophotometer according to Lichtenthaler and Wellburn [41] with slight modifications. Photosynthetic pigments were extracted from 500 mg of leaves with 10 mL of 80% (*v*/*v*) of acetone, centrifuged at 8000 rpm. The OD of the supernatant was recorded at 645 nm, 663 nm and 450 nm. Similarly, the protein content in leaves was determined spectrophotometrically, following the procedure of Zhang et al., [42] with some modification. The supernatant of the homogenized fresh leaves of *Zea mays* (500 mg) in PBS (pH 7.5) was mixed with alkaline Na_2_CO_3_ and Na-K tartrate (aqu.). To the reaction mixture, an aqueous CuSO_4_·5H_2_O solution and Folin phenol reagent was added and incubated for 30 min. The OD was recorded at 650 nm and the protein content was determined using the BSA standard curve.

Sugar was extracted by homogenizing leaves in dH_2_O, centrifuged at 8000 rpm; to the supernatant, 80% of phenol and 5 mL of concentrated H_2_SO_4_ was added. The OD of the reaction mixture was recorded at 420 nm, and the total soluble sugar was estimated using a standard sugar curve. The amount of proline was determined by following the procedure given by Zhang [43]. Proline was extracted through aqueous sulfosalicylic acid. To the filtrate, acid ninhydrin was added, and the reaction mixture was heated at 100 °C. After cooling in an ice bath, toluene was added to the reaction mixture, and the OD of the toluene-aspired layer was measured at 520 nm.

### 4.4. Activity of Antioxidant Enzymes (POD, APX, CAT and SOD)

Enzymes were extracted from fresh leaves while homogenizing in 15 mL of 0.05N PBS (pH 7.0) with EDTA and PVPP. Homogenate was centrifuged at 80,000 rpm, and the supernatant was used to determine the activity of antioxidant enzymes.

For lipid peroxidase (EC 1.11.1.3), activity was determined using the TBARS assay. Enzyme extract was added to trichloroacetic acid and centrifuged at 4000 rpm. To the supernatant, 0.5% thiobarbituric acid in 2.5 N HCl was added in the water bath at 100 °C for about 30 min, allowed to cool in an ice bath. The OD was recorded at 532 nm and 600 nm against the blank-having reaction mixture, without leaves homogenizing. Ascorbic peroxidase (APX EC1.11.1.11) activity was determined, with some modifications, by following the method of Verma et al. [44]. Enzyme extract was added to 1 mL of 0.2 mM of EDTA, 0.05 mM of PBS and 0.5 mM of ascorbic acid and 1 mL of 2% of H_2_O_2_ at 25 °C: the change in the OD was recorded at 290 nm for 3 min. The activity of ascorbic peroxidase was determined using the extinction coefficient of 2.8 mM cm^−1^ by calculating the amount of oxidized ascorbate. Catalase (CAT EC 1.11.1.6) was measured by adding enzyme extract to 2 mL of PBS (pH 7.4), 0.1 mL of EDTA and 20 mM of H_2_O_2_, and change in the OD of the reaction mixture was recorded at 240 nm for 3 min. The methodology of Wang et al. [18] was followed to determine the activity of superoxide dismutase (SOD EC 1.15.1.1) and peroxidase (POD EC1.11.1.X). For SOD activity, enzyme extract was added to 0.075 mM NBT, 13 mM methionine, 0.002 M riboflavin and 0.1 mM EDTA in PBS (pH 7.6) under a light chamber, and the optical density was recorded after 15 min at 560 nm. Similarly, for POD activity, 1.35 mL of 100 mM MES buffer (pH 5.5) was added to the enzyme extract following the addition of 0.1 mL phenylenediamine and 0.05% H_2_O_2_, and the change in OD value was measured at 485 nm for 20 min. In order to control for random errors, all of the experiments were replicated three times.

### 4.5. Statistical Analysis

The data was analyzed through a OW-ANOVA test for the comparison of multiple population means using the experimental sample data through the Statistix 10 package. All of the figures were plotted in Origin 9.1, where each of the mean values are from triplicate data with a 95% CI (error bars).

## 5. Conclusions

Calcium and phosphorus salts act as precursors for the biomimetic synthesis of apatite nanostructures. The thiourea acts as a surface functionalizing agent, validated through FTIR, X-ray diffraction spectroscopy, SEM and EDX spectroscopy. Osmotic stress delayed the germination and seedling growth of *Zea mays*, whereas the application of TU–ANPs ameliorated plant growth via the conversation of photosynthetic pigments and production of osmolytes. The elevated activities of antioxidant enzymes, such as CAT, SOD, APX and POD, in plants under osmotic stress were also restored via foliar application of TU–ANPs. Seed priming and foliar application with optimal doses of TU–ANPs effectively elevated the tolerance level of the crop to osmotic stress and is therefore recommended.

## Figures and Tables

**Table 1:** Impact of TU–ANPs on vegetative growth parameters of *Zea mays* L. grown under different levels of NaCl-based osmotic stress.

Treatments	Leaf Length (cm)	Leaf Width (cm)	Leaf Fresh wt. (gm)	Leaf Dry wt. (gm)	Leaf Area (cm^2^)	Shoot Length (cm)	Shoot Fresh wt. (gm)	Shoot Dry wt. (gm)	Root Length (cm)	Root Fresh wt. (gm)	Root Dry wt. (gm)
T_1_	38 ^b^	2.3 ^cd^	1.9	0.22	42.10 ^ab^	8.5 ^a^	27.13 ^bc^	0.75	6.2 ^bc^	7.5 ^abc^	0.70
T_2_	35 ^cd^	1.9 ^d^	1.4	0.20	41.89 ^bc^	8.5 ^a^	23.44 ^de^	0.73	6.0 ^cd^	7.2 ^bcd^	0.69
T_3_	31 ^d^	1.8 ^d^	1.3	0.20	40.54 ^bc^	6.1 ^bc^	20.29 ^e^	0.70	5.5 ^d^	7.0 ^bcd^	0.68
T_4_	41 ^b^	3.2 ^abc^	2.0	0.25	41.54 ^bc^	5.5 ^bc^	28.07 ^ab^	0.74	6.8 ^cb^	7.7 ^ab^	0.71
T_5_	40 ^bc^	2.7 ^bc^	1.8	0.21	40.64 ^bc^	5.0 ^c^	26.92 ^bc^	0.70	6.0 ^c^	7.1 ^bc^	0.70
T_6_	39 ^bc^	2.3 ^cd^	1.4	0.20	40.55 ^bc^	4.9 ^c^	21.47 ^de^	0.66	5.8 ^cd^	7.0 ^bc^	0.69
T_7_	40 ^bc^	3.5 ^ab^	2.2	0.26	43.42 ^a^	7.6 ^ab^	28.86 ^a^	0.79	7.0 ^a^	7.9 ^ab^	0.74
T_8_	39 ^bc^	2.6 ^bc^	1.9	0.25	42.78 ^ab^	8.7 ^a^	27.20 ^ab^	0.76	6.8 ^ab^	7.6 ^abc^	0.69
T_9_	31 ^d^	1.9 ^d^	1.5	0.22	40.87 ^bc^	7.1 ^ab^	25.11 ^cd^	0.60	6.1 ^cd^	6.9 ^cd^	0.60
T_10_	51 ^a^	3.8 ^a^	2.5	0.25	43.98 ^a^	6.4 ^bc^	29.79 ^a^	0.80	7.1 ^a^	8.0 ^a^	0.73
T_11_	50 ^a^	2.8 ^bc^	2.0	0.19	40.00 ^bc^	7.0 ^ab^	25.37 ^cd^	0.60	6.2 ^bc^	7.6 ^ab^	0.69
T_12_	49 ^a^	1.6 ^d^	1.2	0.17	39.98 ^bc^	6.1 ^bc^	23.25 ^d^	0.59	6.0 ^cd^	6.5 ^d^	0.59
LSD	5.68	1.12	NS	NS	2.25	2.32	2.61	NS	0.65	0.80	NS

T1, (Hydrotreated + non-saline); T2, (Hydrotreated + 60 mM NaCl); T3, (Hydrotreated + 100 mM NaCl); T4, (1 µg/mL TU–ANPs + Non-saline); T5, (1 µg/mL TU–ANPs + 60 mM NaCl); T6, (1 µg/mL TU–ANPs + 100 mM NaCl); T7, (5 µg/mL TU–ANPs + Non-saline); T8, (5 µg/mL TU–ANPs + 60 mM NaCl); T9, (5 µg/mL TU–ANPs + 100 mM NaCl); T10, (10 µg/mL TU–ANPs + Non-saline); T11, (10 µg/mL TU–ANPs + 60 mM NaCl); T12, (10 µg/mL TU–ANPs + 100 mM NaCl).

## Data Availability

All authors have been personally and actively involved in substantial work leading to the paper and will take public responsibility for its content.
